# Synergizing biotechnology and natural farming: pioneering agricultural sustainability through innovative interventions

**DOI:** 10.3389/fpls.2024.1280846

**Published:** 2024-03-22

**Authors:** Anila Badiyal, Rishi Mahajan, Ranbir Singh Rana, Ruchi Sood, Abhishek Walia, Tanuja Rana, Shilpa Manhas, D. K. Jayswal

**Affiliations:** ^1^ Department of Microbiology, Chaudhary Sarwan Kumar Himachal Pradesh Krishi Vishvavidyalaya, Palampur, Himachal Pradesh, India; ^2^ Centre for Geo-Informatics Research and Training, Chaudhary Sarwan Kumar Himachal Pradesh Krishi Vishvavidyalaya, Palampur, Himachal Pradesh, India; ^3^ Department of Agricultural Biotechnology, Chaudhary Sarwan Kumar Himachal Pradesh Krishi Vishvavidyalaya, Palampur, Himachal Pradesh, India; ^4^ Lovely Professional University, Phagwara, Punjab, India; ^5^ National Agricultural Higher Education Project, Indian Council of Agricultural Research, New Delhi, India

**Keywords:** biotechnology, natural farming, resistance, bio-fuels, bio-fortification

## Abstract

The world has undergone a remarkable transformation from the era of famines to an age of global food production that caters to an exponentially growing population. This transformation has been made possible by significant agricultural revolutions, marked by the intensification of agriculture through the infusion of mechanical, industrial, and economic inputs. However, this rapid advancement in agriculture has also brought about the proliferation of agricultural inputs such as pesticides, fertilizers, and irrigation, which have given rise to long-term environmental crises. Over the past two decades, we have witnessed a concerning plateau in crop production, the loss of arable land, and dramatic shifts in climatic conditions. These challenges have underscored the urgent need to protect our global commons, particularly the environment, through a participatory approach that involves countries worldwide, regardless of their developmental status. To achieve the goal of sustainability in agriculture, it is imperative to adopt multidisciplinary approaches that integrate fields such as biology, engineering, chemistry, economics, and community development. One noteworthy initiative in this regard is Zero Budget Natural Farming, which highlights the significance of leveraging the synergistic effects of both plant and animal products to enhance crop establishment, build soil fertility, and promote the proliferation of beneficial microorganisms. The ultimate aim is to create self-sustainable agro-ecosystems. This review advocates for the incorporation of biotechnological tools in natural farming to expedite the dynamism of such systems in an eco-friendly manner. By harnessing the power of biotechnology, we can increase the productivity of agro-ecology and generate abundant supplies of food, feed, fiber, and nutraceuticals to meet the needs of our ever-expanding global population.

## 1 Introduction

The term “sustainability” finds its origin from the Latin word “Sustinere”, which denotes the enhancement of environmental quality and the resource base that can uphold and endure future societal development. The term “sustainable” was used for the first time at the United Nations Conference on Human Environment, Stockholm in 1972 focusing on the preservation of environment for the benefit of human beings across the globe. The major outcome of the Stockholm Conference (1972) was the establishment of the United Nations Environment Programme (UNEP), which became the leading global environmental authority for setting the global environmental agenda. Later on in 1992 in Rio de Janeiro, Brazil, the UN General Assembly called for the United Nations Conference on Environment Development (UNCED) commonly known as the Rio Summit or Earth Summit, 1992 with primary goals of socio-economic development while preventing environmental deterioration (Grubb et al., 2019). A number of multilateral environmental agreements have taken place since 1992. However, the global environment has continued to suffer in terms of loss of biodiversity, desertification, and increasing natural disasters.

Over the past two decades, there has been a growing concern about the need for sustainable agriculture to address the food and fiber requirements of society while also providing enduring solutions for both present and future generations. A fundamental prerequisite for sustainable agriculture is to guarantee social equity and economic viability for farmers and all individuals engaged in agriculture and its associated enterprises. This will encourage them to maintain a healthy environment and support the development of climate-resilient agriculture. One of the popular approaches toward sustainable agriculture is natural farming, popularly known as Zero Budget Natural Farming (ZBNF). The Indian civilization thrived on natural farming for ages and India was one of the most prosperous countries in the world. Traditionally, the entire agriculture was practiced using natural inputs where the fertilizers, pesticides, etc. were obtained from plant and animal products. This continued till the advent of colonial rule in India, which introduced plantation agriculture and turned the focus of farmers from self-sufficient crops to cash crops like indigo, jute, tea, and tobacco. Furthermore, the burgeoning population, the pressure to grow cash crops, and drastic climatic calamities led to the shift of the farming sector toward high-input agriculture.

**Figure 1:**
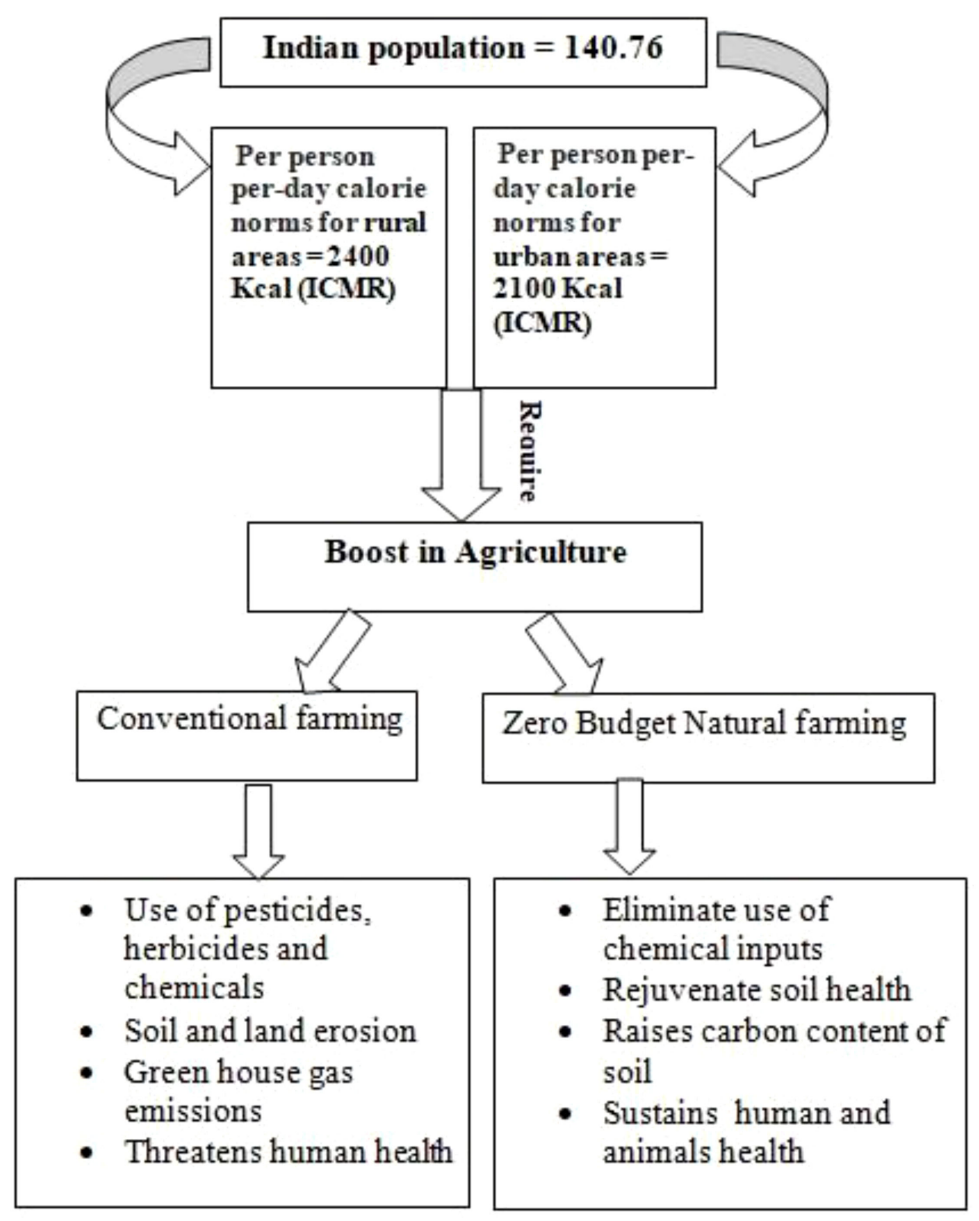
Catalyzing sustainable growth through Zero Budget Natural Farming for India’s burgeoning population.

The concept of natural farming was regained by the Japanese scientist Fukuoka in the 1970s through his book *The One Straw Revolution: An Introduction to Natural Farming*, in which he mentioned it as a do-nothing technique. The concept of natural farming revolves around the idea of self-sufficiency of the natural ecosystem without much human intervention. In India, Padma Shri recipient Mr. Subhash Palekar became the first to adopt the ZBNF system in the 1990s. His concern with the increasing indebtedness and suicide among farmers in India due to the increasing costs of fertilizers and pesticides and their long-term devastating effects on the environment compelled him to advocate the use of low-input technologies in agriculture that should be available within farmlands. He started the natural farming concept in Karnataka and subsequently converted over 50 lakh farmers into practicing ZBNF in various states of India. This method promotes soil aeration, minimal irrigation, intercropping, bunds, and topsoil mulching with crop residue and strictly prohibited intensive irrigation like flooding and deep ploughing tillage practices. However, these traditional practices will not be sufficient to provide food to the estimated 9.7 billion population in 2050. Recently, the Indian Council of Medical Research (ICMR) has set guidelines for per person per day calorie intake to achieve nutritional sufficiency (Chellamuthu et al., 2021). Incorporating modern biotechnological techniques into agriculture is the prerequisite to attaining this goal and mitigating the climate crisis (**Figure 1**).

However, adopting biotechnology in natural farming system is not that easy. There exists an ideological war between natural farming and biotechnology-assisted farming, leading to complete incompatibility among these two systems (Purnhagen and Wesseler, 2021).

Biotechnology in agriculture encompasses a diverse range of techniques, which may include traditional breeding methods that modify living organisms or their components to create or enhance products, improve plants or animals, or engineer microorganisms for particular agricultural applications. It is not exclusive but includes the tools of genetic engineering. It has emerged as a promising tool for crop improvement and led to significant enhancement in agricultural productivity in the 21st century through agricultural revolutions. Within the Indian biotech sector, agricultural biotechnology stands as the third largest segment (as reported by Business Standard in 2013). It is widely recognized as a pivotal sector that plays a significant role in driving the socio-economic development of the country (ABLE INDIA, 2013; Shukla et al., 2018; Lima, 2022). A new biotechnological revolution is estimated to revolve around deciphering the gene codes of living beings leading to “gene revolution”.

Biotechnology often carries a perplexing association with industrial, commodity-based farming, monoculture practices, the extensive use of pesticides, and patented seeds. However, the most significant misinterpretation lies in conflating biotechnology—a production process—with an inherently unsafe and perilous product. This misperception forms the foundation of the stringent regulatory framework that many countries apply to biotech crops.

The current review seeks to advocate the idea that integrating biotechnology with natural farming can offer a promising solution to address key challenges in achieving sustainable agriculture. These challenges include the need to produce sufficient food within the constraints of limited arable land and finite resources, particularly in the face of stresses like drought, salinity, high temperature, and diseases. The aim is to achieve these goals while reducing reliance on synthetic fertilizers and pesticides.

## 2 Strategies for natural farming/eco-agriculture

McNeely and Scherr (2001) have outlined six approaches to achieve the desired outcomes from natural farming. These are stated below:

Participation of local farmers for the creation of bio-diversity reserves. In Wayanad, Kerala, India, a “model” farm has been developed involving local farmers for the cultivation of a diversity of spices, medicinal plants, cash crops, and wild yet economically important trees (*Syzygium travancorium* and *Cinnamomum malabatrum*). The fauna in this farm consists of farm animals, honeybees, and fish. The economic sustainability of the farm is guaranteed by the consistent revenue generated from a diverse array of crops including medicinal, agricultural, and plantation crops as well as through the management of farm animals.

i. Using traditional practices of controlling pests, rain water harvesting, and soil health management using least external inputs have enabled the self-sustainability of the farm. Development of such modal farms will not only reinforce agricultural productivity but also promote the wellbeing of the ecosystem, thus helping conservation naturally.
ii. Integrating cultivated areas with natural habitats to preserve high-quality wildlife environments that are compatible with farming.
iii. Mitigating or even reversing the conversion of wild lands into agricultural use by increasing farm productivity.
iv. Minimizing agricultural pollution through the implementation of more resource-efficient methods for managing nutrients, pests, and waste.
v. Enhancing the quality of habitats in and around farms through the careful management of soil, water, and vegetation resources. Notably, the “biodiversity-rich hotspot” in Orissa, India serves as an excellent example of this approach. On the global scale, “Equator Initiative” is a worldwide movement committed to identifying and supporting innovative partnerships that alleviate poverty through the conservation and sustainable use of biodiversity.

## 3 Biotechnological interventions in natural farming

**Figure 2:**
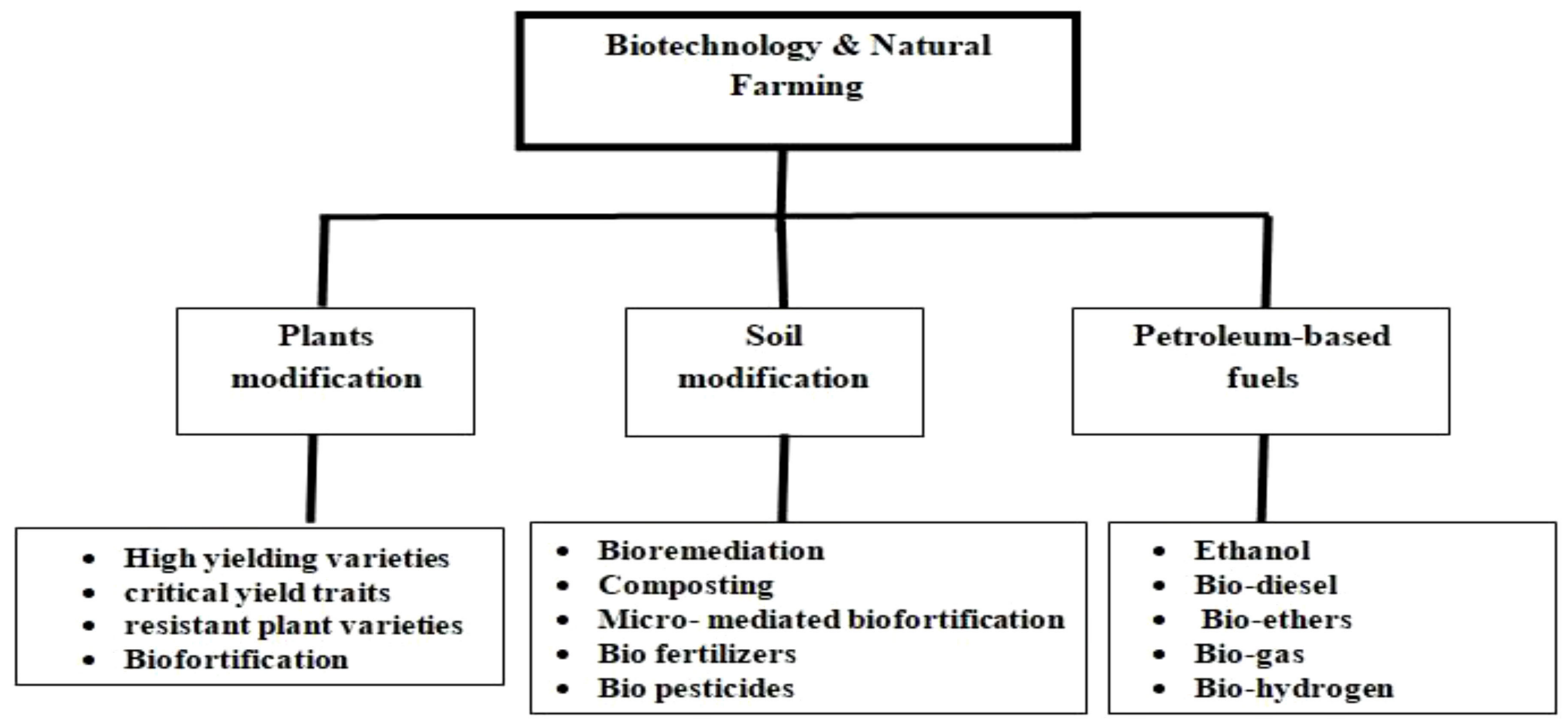
Various approaches for integrating biotechnological tools in natural farming system.

Biotechnology identifies and addresses multifarious aspects of agriculture, leading to a sustainable way of improving the overall productivity of agro-ecosystems. However, we can broadly classify the aspects into three major criteria: modifying plants, modifying the soil, and development of alternatives to fuel inputs for agricultural equipments (**Figure 2**). These aspects have been discussed in detail in the review.

### 3.1 Modifying plants

Conventional plant breeding and selection techniques take much time (six to seven generations) and effort to develop plants with desirable traits. However, when supplemented with novel biotechnological tools like genetic engineering, molecular biology, and micro-propagation, such techniques may result in desirable and stable genotypes within two to four generations (**Table 1**).

**Table 1:** Some examples of successful utilization of biotechnological tools for improving plants.

S. no.	Name of plant	Trait	Candidate gene	Technique used	Reference
1.	Maize	Drought tolerance	*ARGOS8*	CRISPR/Cas9	Shi et al., 2017
		Herbicide tolerance	*IPK1*	ZFN	Shukla et al., 2009; Sedeek et al., 2019
		Northern leaf blight and southern leaf blight	*GST*, *Htn1, pan1, remorin*	Cloning	Ahangar et al., 2022; Wani et al., 2022; Yang et al., 2017
		Head smut	*ZmWAK*		Wani et al., 2022; Yang et al., 2017
		Maize leaf blight and ear mold	*Hm1*		Wani et al., 2022; Yang et al., 2017
		Quality protein	opaque2, *vte4*, *crtRB1*	Marker-assisted backcrossing and selection	Hossain et al., 2023
		Phytic acid content	ZmIPK	CRISPR/Cas9, TALEN	Liang et al., 2014; Sedeek et al., 2019
		Drought	betA, TsVP, CSPs, TPP	Overexpression	Wei et al., 2011
2.	Wheat	Armyworm	*Myc transcription factor 7, Methylesterase 7, Polcalcin Phlp 7-like, Alkaline alpha galactosidase 3, Probable galactinol-sucrose*	Cloning	Hafeez et al., 2021
		Resistance to Stem rust (*Puccinia graminis* f. sp. tritici)	More than 63 genes including *Sr13, Sr21, Sr22, Sr31, Sr35, Sr45, Sr46, Sr50, Sr59, Sr60*	Wide hybridization. Backcrossing and MAS (STS, KASP)	Yazdani et al., 2023; Bouvet, 2022
		Resistance to stripe rust (*Puccinina*)	More than 80 genes including *Yr15, Yr45, Yr61, Yr81-83*	Wide hybridization. Backcrossing and MAS	Yang et al., 2023; Li J. et al., 2020
		Yield-related traits (1,000 kernel weight, spike length, spike compactness, flowering time)	*TaTAP46, TaSDIR1, QGw4B.4* QSc/Sl.cib-5A, QSc/Sl.cib-6A *FT-D1, TaCol-B5*	MAS (CASP, dCASP, STARP, KASP)	Song et al., 2023; Liu H. et al., 2022; Chen L. et al., 2022
		Grain quality (protein content, pre-harvest sprouting tolerance)	GPC, *Glu-D1*,	KASP, SSR	Jiang et al., 2021; Song et al., 2023; Rai and Han, 2023
		Heat, cold drought	*TaFER-5B, TaPYL4, ZmPEPC*	Overexpression	Ayadi et al., 2019; Zhang et al., 2019
			*TaERF3, TaDREB2*	CRISPR	Kim et al., 2018
3.	Rice	Rice blight	*Xa3, Xa4, Xa5, Xa7, Xa10, Xa13, Xa21, Xa23, Xa33, Xa38, Xa40*, and recessive genes	MAS (SSR)	Chukwu et al., 2020; Hsu et al., 2020; Fiyaz et al., 2022
		Grain size and weight	*GS3, Gn1a, GW2, GW5, TGW6, DEP1*	CRISPR/Cas9	Sedeek et al., 2019
		Drought tolerance	*OsPYL9, OsERA1, OsDST*	CRISPR	Ogata et al., 2020; Usman et al., 2022; Santosh Kumar et al., 2021
4.	Oilseed crops like sunflower, soybean, safflower	Oleic acid content	*FAD2*	Mutation breeding	Schuppert et al., 2006; Cao et al., 2013; Msanne et al., 2020
5.	Safflower	γ-Linolenic acid (GLA)	*Δ6DES*	Transgene expression	Nykiforuk et al., 2012
6.	Sorghum	Tiller number, grains per panicle, grain weight	*Bmr2, bmr12, SbSWEET4-3, SbVIN1, SbTST1, SbTST2*	MAS	Zhang et al., 2015; Somegowda et al., 2022
		Plant height	*Dw1, Dw2, Dw3*, and *Dw4*	MAS	Hilley et al., 2016
		Grain quality	*Sh1, SbWRKY, qGW1, KS3*	GWAS and MAS	Kimani et al., 2020
		Flowering and height	*MSD1, MSD3, y1, Wx,DGAT1, AMY3*	GWAS	Rhodes et al., 2017; Dampanaboina et al., 2019
7.	Cherry	Size	*FW2.2/CNR*, Auxin response, cell differentiation, pectin biosynthesis	Bi-parental mapping, association mapping	De Franceschi et al., 2013; Liu Z. et al., 2022
8.	Grapes	Weight	*Aux/IAA9*, DELLA protein	Bi-parental mapping, association mapping	Razi et al., 2020; Doligez et al., 2013; Ban et al., 2016
9.	Logan	Weight	*FW2.2/CNR*, *P450, EXP4*	Bi-parental mapping,	De Mori and Cipriani, 2023
10.	Walnut	Weight, size	Beta-galactosidase, *RBK1*, *BEL1*-like	Association mapping	Bernard et al., 2021

#### 3.1.1 High-yielding varieties

Intergeneric and interspecific hybridization followed by marker-assisted selection (MAS) enabled the development of semi dwarf high-yielding varieties, thus marking the advent of green revolution. Molecular biologists have identified the candidate genes influencing plant height, spike length, seed characteristics, and number of spikelets in wheat (Albahri et al., 2023; Jiang et al., 2023), as well as *DREB* (dehydration-responsive element binding) genes associated with photosynthesis, nitrogen utilization and flowering in rice (Ikeda et al., 2001; Chandler et al., 2022; Wei et al., 2022), male sterility, albino phenotype, and number and weight of kernels in maize (Chen et al., 2018; Kelliher et al., 2019). Characterization and manipulation of such genes can help transfer of these into locally adapted high-yielding cultivars by hybridization followed by MAS or by genome editing technologies.

#### 3.1.2 Enhancing physiological efficiency of plants

Genetic manipulation offers the potential to enhance critical yield-determining traits in plants, including photosynthesis, shoot-to-root biomass ratio, inflorescence architecture, stomatal regulation, nutrient acquisition, and utilization efficiency. One effective strategy for assessing and improving photosynthetic efficiency in plants involves the examination and manipulation of key enzymes. Rubisco, a pivotal enzyme responsible for converting atmospheric CO_2_ into biomass and a significant player in the global carbon cycle, has been a prime target for enhancing crop production. Methods to boost Rubisco activity encompass enhancing the enzyme’s carboxylation capacity, reducing its oxygenation rates through genetic modification, and introducing the complete carbon-concentrating mechanism from cyanobacteria into crop plants via genetic engineering to enhance their photosynthetic capabilities (Hines et al., 2021; Iñiguez et al., 2021). As an example, incorporating Rubisco activase from thermophilic cyanobacteria into plants sensitive to high temperatures has shown promising results in improving crop yield by enhancing photosynthesis under elevated temperature conditions (Ogbaga et al., 2018).

Enhancing photoprotection in plants holds promise for increasing crop yield. Plants have evolved mechanisms to dissipate excess sunlight, safeguarding themselves from damage, albeit at the expense of photosynthetic efficiency (Kromdijk et al., 2016). Research into genes associated with non-photochemical quenching, such as PsbS, has revealed that modifying their expression levels can bolster photoprotection, consequently improving photosynthetic efficiency (Murchie et al., 2015). Likewise, optimizing a plant’s nitrogen use efficiency (NUE) involves modulating nutrient absorption, allocation, and metabolism. Employing biotechnology to manipulate key genes governing nutrient uptake and utilization efficiency is an effective strategy for creating enhanced crop varieties. Genes such as Ammonium transport (AMT), nitrate transport (NRT), glutamine synthetase (GS), and glutamate synthase (GOGAT) play pivotal roles in nitrogen metabolism. Studies have demonstrated that transgenic crops overexpressing these genes exhibit elevated tissue nitrogen levels, increased amino acids, and enhanced biomass and greater seed production (Curatti and Rubio, 2014). For instance, the gene OsDREB1C, responsible for promoting nitrogen use efficiency and resource allocation while shortening growth, has led to a substantial increase in rice yield, ranging from 41.3% to 68.3% compared to wild types when overexpressed (Wei et al., 2022).

#### 3.1.3 Development of resistant plant varieties

**Insect resistance:** The development of insect-resistant transgenic plants stands as a remarkable achievement in agricultural biotechnology, with extensive research efforts carried out by both public and private institutions. The introduction of heterologous DNA is commonly accomplished through genetic transformation methods mediated by *Agrobacterium tumefaciens*, biolistic techniques, or a combination of both (Tabashnik et al., 2013; Carrière et al., 2015). Among the most widely commercialized transgenic crops is cotton, which incorporates *cry* genes sourced from *Bacillus thuringiensis* (Sanahuja et al., 2011). This innovation has proven highly effective in conferring insect resistance (Tabashnik et al., 2013; Carrière et al., 2015). Furthermore, various other notable examples of introducing and expressing foreign genes in crop plants include *API* (arrowhead proteinase inhibitor) in wheat, tobacco, and tomato; *OC-I* (cysteine proteinase inhibitor: *oryzacystatin*-I) in rice; *Vgb* (*Vitreoscilla hemoglobin*) in maize and tobacco; *SacB* (*levansucrase*-encoding gene) in tobacco, rye grass, and tobacco; *JERF*-36 (Jasmonic ethylene-responsive factor) in poplar trees; *BADH* (*betaine aldehyde dehydrogenase* gene) in tobacco, maize, and tomato; and *NTHK1* (*Nicotiana tabacum histidine kinase*-1) in tomato and apple (Tabashnik et al., 2008; Wang et al., 2018). Specifically, transgenic plants like cotton (*Gossypium hirsutum*), soybean (*Glycine max*), and maize (*Zea mays*) have demonstrated resistance to lepidopteran and coleopteran larvae (caterpillars and rootworms), leading to substantial reductions in pesticide usage and production costs, all while enhancing crop yields.

**Disease resistance:** Modifying host–pathogen interactions, signaling mechanisms, and associated proteins has led to the development of disease-resistant crop varieties. In wheat, the cloning and utilization of several adult plant resistance (APR) genes have enabled the creation of transgenic lines resistant to rust and powdery mildew pathogens at both seedling and adult stages (Krattinger et al., 2009; Risk et al., 2013; Ellis et al., 2014). The introduction of the *Lr34* allele, which codes for resistance against leaf rust, into various crops such as rice, barley, sorghum, maize, and durum wheat, as well as Lr67 into barley, has conferred resistance to a wide range of biotrophic pathogens (Risk et al., 2013; Krattinger et al., 2016; Sucher et al., 2017). Advanced techniques like Targeting Induced Local Lesions in Genomes (TILLING) and genome-editing methods such as Zinc Finger Nucleases (ZFN), Transcription Activator-Like Effector Nucleases (TALENs), and notably Clustered Regularly Interspaced Short Palindromic Repeats (CRISPR) and Crisper-associated protein (Cas) have become powerful tools in functional genomics and crop breeding. Simultaneous modification of the three homeologs of EDR1 in wheat has resulted in powdery mildew-resistant plants (Zhang Y. et al., 2017). Moreover, rice lines with broad-spectrum resistance to *Xanthomonas* have been created by editing the promoter regions of SWEET11, SWEET13, and SWEET14 genes (Xu et al., 2019). Powdery mildew resistance has been achieved through editing MLO (Mildew Resistance Locus) in various plant species, including wheat (Wang et al., 2014; Acevedo-Garcia et al., 2017), tomato (*S. lycopersicum*) (Nekrasov et al., 2017), and grapevine (*Vitis vinifera*) (Wan et al., 2020).

**Herbicide resistance:** Weeds are a persistent issue in agriculture, hindering crop growth by competing for essential resources like water, nutrients, sunlight, and space. They also act as carriers for various insects and harmful microorganisms. Uncontrolled weed growth can significantly reduce crop yields, leading farmers to use methods like herbicides containing glyphosate and glufosinate, tilling, and manual weeding to manage them. Glyphosate herbicides work by inhibiting the EPSPS enzyme, vital for producing aromatic amino acids, vitamins and other plant metabolites. However, these methods can lead to problems like groundwater contamination and environmental damage, causing declines in plant and animal species (Mazur and Falco, 1989; Powles, 2018). Biotechnological advancements have given rise to herbicide-resistant crop varieties, such as those tolerant to glyphosate and glufosinate (Tan et al., 2006). These crops are engineered with genes like *CP4-EPSP synthase* and *GOX* (*glyphosate oxidoreductase*), which produce glyphosate-tolerant EPSPs and glyphosate-degrading enzymes (Shaner, 2000; Owen and Zelaya, 2005).

**Abiotic stress resistance:** The advancement of functional omics and computational biology software and tools has enabled the identification of candidate genes responsible for abiotic stress (AbS) from diverse gene pools. Techniques like RNA-Seq, random and targeted mutagenesis, gene shifting, complementation, and synthetic promoter trapping are valuable for analyzing AbS-responsive genes and understanding tolerance mechanisms, including post-translational modifications (PTM), protein degradation, and interactions with non-coding miRNA (Chantre Nongpiur et al., 2016). Genome-wide association studies (GWAS) have gained popularity for discovering and characterizing stress-responsive genes, which, when introduced into crop plants, enhance their tolerance to various AbS conditions (Le et al., 2021). Chan et al. (2006) reported a total of 13,022 AbS-related ESTs from *Hordeum vulgare*, 13,058 genes from *Oryza sativa*, 17,189 from *Sorghum bicolor*, 2,641 from *Secale cereale*, 20,846 from *Triticum aestivum*, and 5,695 regulators from *Z. mays* using the gene index of the TIGR database (http://www.tigr.org/tdb/tgi/) (Chan et al., 2006). Identifying these ESTs and incorporating them into widely cultivated elite cultivars through *in vitro* mutagenesis, genetic transformation, tissue culture, and MAS using omics tools have resulted in the development of several abiotic stress-tolerant plant varieties (Cassia et al., 2018). However, discovering and maintaining ESTs in a crop is very tedious and time-consuming as compared to maintaining cDNA libraries of the transcribed loci, the majority of which come from DREB/CBF, ERF, NAC, D-ZipI, and WRKY families (Noor et al., 2018; Jeyasri et al., 2021). Additionally, recent research has identified and dissected the QTLs for plant height, spike length, and seed characteristics in recombinant inbred lines by combining linkage mapping and weighted gene co-expression network analysis (WGCNA) (Villalobos-López et al., 2022; Wei et al., 2022).

#### 3.1.4 Bio-fortification

“Bio-fortification,” also known as “biological fortification,” involves enhancing the nutritional value of food crops by increasing nutrient availability to the consumer population, utilizing modern biotechnology techniques, conventional plant breeding, and agronomic practices (Malik and Maqbool, 2020; Shahzad et al., 2021; Krishna et al., 2023).

Bio-fortification can be achieved by following various conventional approaches like intercropping and mixed cropping or by utilizing biotechnology in modifying rhizosphere of the crops. Intercropping or mixed cropping of cereals along with legumes employs complementation (partitioning resources or reducing competition between species) and facilitation (positive interaction between the species leading to enhanced growth, reproduction, and survival of both) as the major ecological phenomena leading to improved resource use efficiency. Complementarity of nutrient uptake (N, P, Fe, and Zn) in cereal–legume mixed-cropping/intercropping systems provides a unique advantage for the system to be sustainable in the long run (Dissanayaka et al., 2021; Ebbisa, 2022). Furthermore, plant-growth-promoting microorganisms (PGPMs) enhance the bioavailability of nutrients like P, K, Fe, Zn, and Si to plant roots through chelation, acidification, decomposition of organic matter, and suppression of soil-borne pathogens and can replace inorganic fertilizers and pesticides (Maitra and Ray, 2019; Karnwal, 2021).

Bio-fortification is a socially, economically, and environmentally sustainable approach, especially in developing countries, as compared to alternative fortification strategies. To date, staple crops like rice, wheat, maize, sorghum, and vegetables such as common bean, potato, sweet potato, and tomato have been fortified through genetic manipulation, conventional breeding, and agronomic methods. Cassava, cauliflower, and banana have undergone bio-fortification using both transgenic and breeding techniques, while barley, soybean, lettuce, carrot, canola, and mustard have been bio-fortified through transgenic and agronomic approaches. Transgenic-based approaches offer the advantage of targeting multiple crops once a beneficial gene is identified. Notable successful examples of transgenically fortified crops include high-lysine maize, high-unsaturated-fatty-acid soybean, high-pro-vitamin A and iron-rich cassava, and pro-vitamin A-rich Golden rice. Golden rice, in particular, marked a significant breakthrough with the potential to combat vitamin A deficiency (Burkhardt et al., 1997; Ye et al., 2000; Beyer et al., 2002; Datta et al., 2003; Paine et al., 2005).

### 3.2 Modifying soils

#### 3.2.1 Bioremediation

Bioremediation is a process that primarily harnesses microorganisms, plants, or microbial/plant enzymes to detoxify and degrade contaminants in various environments. In modern crop production, xenobiotics are predominantly organic compounds that do not readily break down naturally. As a result, their accumulation in the environment can lead to their entry into the food chain and water resources, posing risks to the health of animals and humans (Germaine et al., 2006; Chen et al., 2011). Plant–microbe associations, such as plant–endophytic or plant–rhizospheric partnerships, offer potential for enhancing nutrient uptake and the degradation of organic pollutants, thereby contributing to environmental restoration (Zhang et al., 2017).

Bioremediation of complex hydrocarbons can be through natural attenuation/intrinsic bioremediation (using indigenous microflora for decomposing pollutants), bioaugmentation (applying potential microbes for faster decomposition), bio-stimulation (modifying the microenvironment for facilitating microbial action), and surfactant-assisted biodegradation (Kebede et al., 2021).

Furthermore, rhizosphere microorganisms can be used to remove heavy metals from soils through biosorption (adsorption of heavy metals on the cell wall constituents, i.e., carbohydrates, proteins, and teichoic acids of microorganisms), bioaccumulation (accumulation of heavy metals inside the cytoplasm through an import-storage system mediated by metal transporter proteins), bioleaching (solubilizing metal sulfides and oxides from ore deposits and secondary wastes), biomineralization (conversion of complex metal ions into carbonates, sulfates, oxides, phosphates, etc. through metabolic pathways), and biotransformation (alteration of metal complexes into those with more polarity to make them water soluble) (Tayang and Songachan, 2021).

Examples of successful utilization of microorganisms for biosorption of complex hydrocarbons include removal of lead and cadmium by *Staphylococcus hominis* strain AMB-2 (Rahman et al., 2019); and cadmium, lead, and copper by fungi *Phanerochaeta chrysosporium* (Say et al., 2001), *Spirulina platensis, Chlorella vulgaris, Oscillatoria* sp., and *Sargassam* sp. (Leong et al., 2021). Bioaccumulation has been shown in *Pseudomonas putida* 62 BN (Rani et al., 2013), *Bacillus cereus* M116 (Naskar et al., 2020), and fungi *Monodictys pelagic* and *Aspergillus niger* (Sher and Rehman, 2019). Researchers have shown that bioleaching by microorganisms is an economic as well as eco-friendly approach toward efficient extraction of metals gold, cobalt, copper, uranium, zinc, etc. from low-grade ores (Tayang and Songachan, 2021). Even arsenic bioleaching has been possible with *Acidithiobacillus ferrooxidans* and *Acidithiobacillus thiooxidans* (Zhang and Gu, 2007). Metal immobilization through biomineralization of metals from *Bacillus* sp (Zhang et al., 2019), *Acinetobacter* sp., and *Micrococcus* sp. oxidized toxic As(III) into harmless and less soluble As(III) and decreased its toxicity, as shown by Nagvenkar and Ramaiah (2010).

Rhizoremediation can bolster phytoremediation by promoting the growth of microbial communities and their associated activities, facilitated by root exudation, turnover, and the possible induction of enzymes responsible for degradation due to the secretion of secondary metabolites in plants (Didier et al., 2012). Certain common garden and ornamental plants, including *Glandularia pulchella*, *Aster amellus*, *Portulaca grandiflora*, *Petunia grandiflora*, and *Zinnia angustifolia*, have been recognized for their capacity to degrade pollutants and dyes (Khandare and Govindwar, 2015) and effectively remove polychlorinated biphenyls from the soil (Erdei, 2005; USEPA, 2005; Erakhrumen and Agbontalor, 2007; Passatore et al., 2014; Kurade et al., 2021).

Notably, *Typha domingensis*, in combination with xenobiotics effluent-degrading endophytic bacteria, achieved a substantial improvement in the removal of parameters like biochemical oxygen demand (BOD) (77%), chemical oxygen demand (COD) (79%), total suspended solids (TSS) (27%), and total dissolved solids (TDS) (59%) (Shehzadi et al., 2014). An efficient plant–bacterial synergistic system has been employed for treating substantial volumes of xenobiotic effluents in wastewater wetlands (Kabra et al., 2013) (**Table 2**).

**Table 2:** Some examples of use of biotechnologically modified microbial formulations in agriculture.

S. no.	Trait	Microorganisms involved	Technique used	References
1.	Nutrient solubility, crop yield of soybean	*Pseudomonas* spp., *Bacillus* spp., *Klebsiella* spp., *Aspergillus* spp., and *Azotobacter* spp.	Liquid bio-inoculant based on sugar and coconut water	Neneng, 2020
2.	Seed germination in Capsicum	*Serratia liquefaciens* CPAC53, *S. plymuthica* CPPC55, *P. tolaasii* P61, and *P. yamanorum* OLsSf5	Encapsulation of biofertilizers	Quiroz-Sarmiento et al., 2019
3.	Ca alginate Diuron herbicide degradation	*Delftia acidovorans* and *Arthrobacter*	Immobilization	Bazot and Lebeau, 2009
4.	Heavy metal bioremediation	*Cronobacter muytjensii* KSCAS2	Biosorption	Saranya et al., 2018
5.	Lead and cadmium bioremediation	*Monodictys pelagic* and *Aspergillus niger*	Bioaccumulation	Sher and Rehman, 2019
6.	Arsenic bioremediation	*Acidithiobacillus ferrooxidans* and *Acidithiobacillus thio-oxidans*	Bioleaching	Zhang and Gu, 2007
7.	Bioethanol (1-butanol, isobutanol, and isopentanol as ethanol substitutes) production	Yeast (*Saccharomyces cerevisiae*), *Clostridium thermocellum*	Engineering fermentative pathways, non-fermentative keto acid pathways, and isoprenoid pathways	Lane et al., 2020
8.	Hydrogen production	*Caldicellulosiruptor*	Engineering glycolytic pathway	Cha et al., 2013

#### 3.2.2 Restructuring soil through composting

Manure fertilization is a sustainable practice by turning harmful waste into a bioavailable resource. However, improper management can also lead to serious eco-environmental concerns through release of pathogens, toxic micro-pollutants, greenhouse gases, and nuisance odors. Composting, the process of decomposition of complex waste organic matter into the simpler readily assimilable biomolecules, is a sustainable way to address the aforesaid problem but is limited by a slow rate (Gautam et al., 2012; Singh et al., 2021). The microorganisms effectively contributing toward composting include fungi (*Ascomycetes*, Fungi imperfecti, Basidiomycetes, *Trichoderma*, and *Phanerochaete*), bacteria (*Bacillus* spp., *Cellulomonas, Cytophaga*, and *Sporocytophaga*), and actinomycetes (*Thermoactinomyces, Streptomyces, Micromonospora*, and *Thermomonospora*). The process of composting is mediated by extracellular production of laccase, which facilitates humification and polymerization in livestock manure. Genetically engineered microbes that produce large amounts of extracellular laccase not only enhance the fertilizer quality of end products but also manage their eco-environmental risks by inactivating pathogens, detoxifying micro-pollutants, and stabilizing organic nutrients, but the process is quite fast, thus preventing the loss of C and N into environment (Jiang et al., 2021; Niu et al., 2021).

#### 3.2.3 Microbe-mediated bio-fortification

There are vitamins and minerals that are required in the human body in trace amounts, but their deficiency is manifested as several physiological disorders. Many of such vitamins and minerals are not even synthesized by plants. A good example is Vitamin B12, which cannot be synthesized by plants; hence, bio-fortification of this vitamin can be achieved by the help of microbes like bacteria and archea in the plant rhizosphere (Ku et al., 2019; Krishna et al., 2023). Phyto-stimulation by plant growth-promoting rhizobacteria (PGPRs) benefits the plants by increasing the nutrient availability (Kaur et al., 2020; Chouhan et al., 2021). Recent research has identified the contribution of PGPRs in the bio-fortification of iron, zinc, selenium, and other elements in several crops (Kaur et al., 2020; Singh and Prasanna, 2020; Mushtaq et al., 2021; Khanna et al., 2023).

#### 3.2.4 Bio-fertilizers

Bio-fertilizers are formulations containing live microbes that contribute to soil fertility enhancement by nitrogen fixation from the atmosphere, phosphorus solubilization, and decomposition of organic matter. This improves nutrient bioavailability and accessibility to plants, leading to enhanced growth and productivity (Okur, 2018; Abbey et al., 2019). Utilizing bio-fertilizers offers several advantages, including cost-effectiveness, increased nutrient availability, improved soil health and fertility, protection against soil-borne pathogens, enhanced tolerance to biotic and abiotic stress, and reduced environmental pollution (Chaudhary et al., 2021; Chaudhary et al., 2022a). Researchers may follow diverse approaches like cultivation on selective media, metabolic analyses through high-performance liquid chromatography-mass spectrometry (HPLC-MS) and gas chromatography-mass spectrometry (GC-MS), proteomic studies using two-dimensional electrophoresis and matrix-assisted laser desorption and ionization coupled to time-of-flight mass spectrometry (MALDI-ToF/MS), and metagenomic/metatranscriptomic tools for identifying potential plant growth-promoting microbes (Pirttilä et al., 2021). Notable examples of bio-fertilizers include nitrogen-fixing microbes such as *Rhizobium*, *Azotobacter*, *Bacillus*, *Clostridium* (Sumbul et al., 2020; Gohil et al., 2022); phosphorus-solubilizing microbes like *Bacillus*, *Rhizobium*, *Aspergillus*, and *Penicillium* (Zhang et al., 2020); potassium-solubilizing microbes (*Bacillus*, *Clostridium*, and *Acidithiobacillus*) (Ali et al., 2021; Chen R. Y. et al., 2022); sulfur-solubilizing microbes (*Bacillus*, *Beggiatoa*, and *Aquifer*) (Kusale et al., 2021); zinc-solubilizing microbes (*Bacillus*, *Pseudomonas*, and *Serratia*) (Nitu et al., 2020); phytohormone-producing microbes (*B. thuringiensis*) (Batista et al., 2021); siderophore-producing microbes (*Pseudomonas* and *Bacillus*) (Sarwar et al., 2020); organic matter-decomposing microbes (*Bacillus*, *Pseudomonas*, and *Trichoderma*) (Baldi et al., 2021; Galindo et al., 2022); and PGPRs such as *Rhizobium*, *Pseudomonas*, and *Bacillus* (Khati et al., 2018; Chaudhary et al., 2022b). Bio-fertilizer formulation includes the mixture of selected beneficial strain/s with a suitable vehicle that preserves the viability of the microorganisms in either a dormant or metabolically active state during transport, storage, and application (Schoebitz et al., 2013). A successful microbial formulation must overcome the conditions of temperature, humidity, salinity, UV radiation, and water stress present in the soil besides being effective and competitive against the native microbial populations of the soil (Glare and Moran-Diez, 2016). Classically, bio-fertilizers may be formulated and applied in the form of liquid (culture broths or formulations based mainly on water, mineral, or organic oils) or solids (mixing the microorganisms with a solid support, such as vermiculite, perlite, sepiolite, kaolin, diatomaceous earth, natural zeolite, peat, or clay). However, the failure of these to protect the microbes in drastic abiotic conditions has paved the way for introduction of bio-encapsulated microorganisms. The use of encapsulating polymers like alginate, chitosan, gellan gum, gelatine, agar, bentonite, starch, and laponite has proven to be highly effective in increasing the viability of microorganisms by protecting them against the adverse abiotic conditions (Rojas-Sánchez et al., 2022).

#### 3.2.5 Bio-pesticides

Bio-pesticides are naturally occurring compounds or agents derived from animals, plants, and microorganisms, including bacteria, cyanobacteria, and microalgae. They are used for controlling agricultural pests and pathogens. Key advantages of bio-pesticides over chemical pesticides include their eco-friendly nature, target specificity, and non-lethality to non-target organisms. Bio-pesticides are highly effective even in small quantities and break down quickly without leaving problematic residues. They employ multiple modes of action, such as growth regulation, gut disruption, metabolic poisoning, neuromuscular toxins, and non-specific multi-site inhibition (Sparks and Nauen, 2015; Dar et al., 2021). These diverse modes of action against targeted pests reduce the likelihood of resistance development, which is common with chemical pesticides.

Additionally, when microorganisms are used as bio-pesticides in the fields, they not only combat pathogens but also contribute to plant health and soil fertility maintenance through various effects.

Major examples of bio-pesticides include microorganisms like *B. thuringiensis*, *Pseudomonas aeruginosa*, *Yersinia*, and *Chromobacterium* and fungi like *Metarhizium*, *Verticillium*, *Hirsutella*, and *Paecilomyces* (Fenibo et al., 2021). Biochemical pesticides encompass insect pheromones (Ghongade and Sangha, 2021; Singh et al., 2021), plant-based extracts and essential oils (Gonzalez-Coloma et al., 2013; Ujváry, 2001), insect growth regulators (Feduchi et al., 1985; Arena et al., 1995), and genetically modified organism (GMO) products, especially RNAi-based plant-incorporated protectants (PIPs) (Parker and Sander, 2017; Wei et al., 2018; Ganapathy et al., 2021).

However, the wider adoption of biopesticides faces limitations such as high production costs, challenges in meeting global market demands, variations in standard preparation methods and guidelines, determination of active ingredient dosages, susceptibility to environmental factors, and relatively slower action.

### 3.3 Development of alternatives to petroleum-based fuels for agricultural equipments

Presently, a significant number of farmers rely heavily on non-renewable resources like diesel and gasoline to fuel their agricultural equipment. This dependence poses several challenges: (1) the depletion of a finite resource, (2) adverse environmental effects, and (3) vulnerability to unpredictable price fluctuations. Transitioning to biologically derived fuels, commonly known as bio-fuels, such as ethanol or biodiesel, could offer a viable solution. By utilizing crops like maize or soybean for bio-fuel production, farmers may not only insulate themselves from the uncertainties of fuel price hikes but also create an alternative revenue stream. This shift toward bio-fuels aligns with sustainable practices, fostering both economic resilience and environmental stewardship in the agriculture sector.

Bio-fuel is the fuel (solid, liquid, and gaseous) extracted from biomass (living organisms especially plants and microorganisms) (Braun et al., 2008). For the production of bio-fuels, starch-based agrowastes are prominently exploited due to their limited utility for commercial production of animal and human consumables (Nguyen et al., 2010). There are microorganisms that facilitate the production of ethanol, bio-diesel, bio-ethers, bio-gas, syngas, and bio-hydrogen from lignocelluloses degradation and subsequent glucose fermentation. These include *Kluyveromyces marxianus, Clostridium shehatae, Thermoanaerobacter* sp., *Saccharomyces cerevisae, Escherichia coli*, *Zymomonas mobilis*, *Pichia stipitis*, *Candida brassicae, Mucor indicus*, cyanobacteria (*Synechocystis* sp., *Desertifilum* sp., *Synechococcus* sp., *Phormidium corium*, *Synechocystis* sp., *Oscillatoria* sp., and *Anabaena* sp.) (Kossalbayev et al., 2020), and microalgae (*Scenedesmus obliquus*, *Chlamydomonas reinhardtii*) (Martinez-Burgos et al., 2022).

Biotechnology is revolutionizing the production of ethanol from cellulose by harnessing genetically modified yeasts and bacteria, enhancing efficiency and sustainability. However, the major constraints experienced by engineered microbial cell factories include metabolic imbalance as a result of nutrient depletion, metabolite accumulation, evolutionary pressure, genetic instability, or other stress factors. Hence, bio-prospecting (screening native strains isolated from diverse sources for novel and functional enzymes) and analyzing their genome for gene of interest and metabolome for possible alternate pathways to enhance the biofuel production can be useful (Kim et al., 2002; Adegboye et al., 2021). Successful examples include production of higher octane hydrocarbons (substitutes to ethanol such as 1-butanol, isobutanol, and isopentanol with improved fuel qualities), through engineering fermentative pathways, non-fermentative keto acid pathways, and isoprenoid pathways (Lo et al., 2017; Adegboye et al., 2021).

Furthermore, genetic engineering plays a pivotal role in developing high energy-yielding plant varieties, surpassing the output of existing strains. Additionally, biotechnological advancements open doors to the conversion of agricultural waste into viable fuel sources, making the most of sustainable resources and minimizing environmental impact.

There are microbes like *Gluconobacter sulfurreducens, Actinobacillus succinogenes*, *Proteus* spp., *Shewanella putrefaciens*, *Rhodoferax ferrireducens*, and *D. desulfurcans*, which facilitate the production of bio-electricity (Ieropoulos et al., 2005; Capodaglio et al., 2013).

## 4 Conventional vs. modern natural farming

Conventional natural farming is basically a do-nothing technique that relies totally on natural inputs for the maintenance of the agro-ecosystem, thus reducing the use of artificial fertilizers and industrial pesticides. Agricultural biotechnology also exploits the natural inputs (microbes, wild relatives of cultivated plants, and agricultural wastes) but amplifies their effects with the application of technology in them. Conventional natural farming requires minimum inputs, hence called ZBNF. On the other hand, biotechnology-assisted natural farming requires financial support in research and development, but once the variety/product is ready to be used in fields, it becomes self-sustainable.

Furthermore, biotechnology is a catalyst for introducing novel concepts, methodologies, products, and procedures essential for problem-solving, particularly addressing the specific requirements of smallholder farmers in developing nations (Thompson, 2008; FAO, 2011; Yuan et al., 2011). Biotechnology-assisted breeding stands out for its unique ability to swiftly integrate advantageous traits from wild crop relatives, enhancing both yield and nutritional benefits. This approach also widens the spectrum of genes in agricultural biodiversity, enhancing crop resilience against pests, diseases, and the impacts of climate change (Asdal, 2008). The heightened efficiency in selection processes significantly accelerates breeding cycles, expediting the introduction of new plant varieties. In contrast, traditional methods often necessitate years to eliminate unfavorable traits and incorporate desired ones with elite germplasm background.

Agricultural biotechnology holds the promise of addressing critical issues in the pursuit of sustainable agriculture. These challenges include the imperative to produce an ample food supply within the constraints of diminishing arable land and finite resources, notably water, all while contending with various environmental stresses like drought, salinity, and heat.

## 5 Impact of biotechnology-assisted natural farming on

**Environmental health:** Biotechnology-derived crops have often been associated with concern regarding their potential impact on species abundance and ecosystem biodiversity. However, the utilization of bio-herbicides, as opposed to chemical herbicides, can lead to a reduction in the population and variety of targeted weeds and weed seeds within agricultural systems, all the while mitigating greenhouse gas emissions (Chamberlain et al., 2007).

Additionally, there have been worries about the loss of diversity within crop species (Gepts and Papa, 2003). Nevertheless, research focusing on cotton and soybean varieties in the USA suggests that the introduction of transgenic varieties had little to no discernible impact on genetic diversity (Bowman et al., 2003; Sneller, 2003). Furthermore, numerous public sector collections of germplasm from cultivated crops and their wild relatives exist with the purpose of preserving genetic diversity.

In comparison to conventional insecticide use, Bt crops demonstrate an ability to conserve non-target species, resulting in increased arthropod abundance and diversity (Devine, 2005; Torres and Ruberson, 2005; Cattaneo et al., 2006). They also facilitate more effective biological control of pests that are not susceptible to Bt toxins (Naranjo, 2005).

The non-restricted movements of beneficial arthropods between different cropping systems can facilitate conservation of non-target species in nearby (non-transgenic) crops (Prasifka et al., 2009). One of the major threats to sustainability is the widespread evolution of resistant pest populations. However, the limited selection pressure on insect populations by insect-resistant crops can delay the phenomenon. Furthermore, incorporating the non-biotech-derived crops known as refuges provides susceptible insects to mate with any resistant individuals emerging from Bt crops, resulting in hybrid progeny that cannot survive on insect-resistant plants (Environmental Protection Agency (EPA), 2001).

**Economic status:** The concept of natural farming is inherently tied to the notion of economic sustainability, emphasizing the need for agricultural practices to be financially viable and capable of generating adequate income to support the livelihoods of farmers and individuals in related sectors (Das et al., 2023). Economic incentives play a pivotal role in driving the widespread adoption of sustainable agricultural practices. Biotechnology-assisted natural farming, for instance, facilitates the efficient implementation of precision agriculture, ultimately leading to cost reduction. The diversification of crops and livestock offers a means to mitigate risks associated with weather extremes, market fluctuations, or disease/pest outbreaks.

Incorporating insect-resistant crops into cropping strategies diminishes the need for expensive chemical insecticides and pesticides. Modified soils aid in water conservation, thereby reducing erosion-induced damage within agro-ecosystems. The preservation of natural resources contributes to the reduction of irrigation costs and enhances long-term productivity.

**Social system:** Agriculture, as a sector deeply rooted in communities, fosters opportunities and collaborative relationships among farming families and community members. Natural farming, which relies on natural inputs and involves substantial human engagement, not only aligns with cultural traditions tied to farming but also safeguards the community’s cultural identity. It acts as an avenue for job creation and wealth generation and spurs economic growth within the community.

## 6 Conclusion

In conclusion, biotechnology in agriculture has emerged as a multifaceted tool that encompasses a diverse range of techniques, ranging from traditional breeding methods to advanced genetic engineering. This comprehensive approach has played a pivotal role in the 21st-century agricultural revolutions, contributing significantly to enhanced productivity and the socio-economic development of countries, with agricultural biotechnology standing as a key segment within the Indian biotech sector. The association of biotechnology with industrial farming practices has led to misconceptions and a stringent regulatory framework in many countries. It is crucial to distinguish between the biotechnological production process and the safety of the end product, addressing the misperception that underlies regulatory challenges. Biotechnology, when applied judiciously, addresses various aspects of agriculture, promoting sustainability in three major criteria: improving plants, modifying soil, and developing alternatives to fuel inputs for agricultural equipment.

The integration of functional omics, computational biology, and advanced techniques like RNA-Seq and GWAS to modify critical agro-morphological traits in plants besides altering host–pathogen interactions, signaling mechanisms, and associated proteins holds promise for disease-resistant high-yielding varieties. These advancements are crucial for addressing contemporary challenges, including climate change and resource constraints, in the pursuit of sustainable agriculture.

As we anticipate a new biotechnological revolution focused on deciphering gene codes and the “gene revolution,” it is imperative to foster a balanced understanding of biotechnology’s potential in synergy with natural farming practices. This synergy holds the key to pioneering agricultural sustainability through innovative interventions, encompassing microbe-mediated bio-fortification, bioremediation, restructuring soil through composting, and developing alternatives to petroleum-based fuels for agricultural equipment. By embracing these innovative approaches, we can pave the way for a sustainable future in agriculture that maximizes productivity while minimizing environmental impact and ensuring food security for generations to come.

In terms of environmental sustainability, genetically engineered crops have proven to be advantageous over conventional insecticides, conserving non-target species, enhancing arthropod abundance and diversity, and promoting more effective biological control of pests. The incorporation of insect-resistant crops not only reduces the need for expensive chemical inputs but also contributes to soil modification for water conservation, decreasing erosion-induced damage and lowering irrigation costs. The implementation of refuges alongside insect-resistant crops serves as a strategic measure to delay the evolution of resistant pest populations, emphasizing the importance of maintaining a balanced ecosystem.

The economic sustainability of natural farming is underscored by its inherent link to financial viability and income generation for farmers. Biotechnology-assisted natural farming facilitates precision agriculture, reducing costs and offering a diversified approach to mitigate risks associated with weather, market fluctuations, and disease/pest outbreaks. On a societal level, the social system surrounding agriculture is positively influenced by the adoption of natural farming practices. The alignment of natural farming with cultural traditions fosters a sense of identity and community resilience. It serves as a source of job creation, wealth generation, and economic growth within the community, reinforcing the interdependence of agriculture with social wellbeing.

In conclusion, the impact of biotechnology-assisted natural farming on environmental health, economic status, and social systems demonstrates the potential for a harmonious integration of technological advancements with sustainable agricultural practices.

## Author contributions

AB: Conceptualization, Investigation, Writing – original draft. RM: Conceptualization, Writing – review & editing. RR: Funding acquisition, Resources, Visualization, Writing – review & editing. RS: Investigation, Writing – original draft. AW: Conceptualization, Writing – review & editing. TR: Writing – review & editing, Methodology. SM: Writing – review & editing, Data curation. DKJ: Resources, Validation, Visualization, Writing – review & editing.
